# Lose-Shift Responding in Humans Is Promoted by Increased Cognitive Load

**DOI:** 10.3389/fnint.2018.00009

**Published:** 2018-03-08

**Authors:** Victorita E. Ivan, Parker J. Banks, Kris Goodfellow, Aaron J. Gruber

**Affiliations:** Canadian Centre for Behavioral Neuroscience, Department of Neuroscience, University of Lethbridge, Lethbridge, AB, Canada

**Keywords:** lose-switch, prefrontal cortex, adult, children, cognitive load, WSLS

## Abstract

The propensity of animals to shift choices immediately after unexpectedly poor reinforcement outcomes is a pervasive strategy across species and tasks. We report here on the memory supporting such lose-shift responding in humans, assessed using a binary choice task in which random responding is the optimal strategy. Participants exhibited little lose-shift responding when fully attending to the task, but this increased by 30%–40% in participants that performed with additional cognitive load that is known to tax executive systems. Lose-shift responding in the cognitively loaded adults persisted throughout the testing session, despite being a sub-optimal strategy, but was less likely as the time increased between reinforcement and the subsequent choice. Furthermore, children (5–9 years old) without load performed similarly to the cognitively loaded adults. This effect disappeared in older children aged 11–13 years old. These data provide evidence supporting our hypothesis that lose-shift responding is a default and reflexive strategy in the mammalian brain, likely mediated by a decaying memory trace, and is normally suppressed by executive systems. Reducing the efficacy of executive control by cognitive load (adults) or underdevelopment (children) increases its prevalence. It may therefore be an important component to consider when interpreting choice data, and may serve as an objective behavioral assay of executive function in humans that is easy to measure.

## Introduction

The ability to adapt behavior in response to dynamic environments is a paramount feature of the mammalian brain. The prefrontal cortex (PFC) is credited as a brain region involved in supporting behavioral adaptation through executive functions such as reasoning, working memory, impulse suppression and outcome evaluation (Kane and Engle, 2002; Balleine and O’Doherty, 2009; Passingham and Wise, 2012). Behavioral flexibility, a broad concept generally embodying the change in response policies to accommodate changing environmental or internal states, is a key feature of the decision making processes that depends on the PFC in humans (Garavan et al., 2002; Braver et al., 2009), non-human primates (Barraclough et al., 2004; Moore et al., 2009), and rats (Kolb, 1984; de Bruin et al., 1994; Ragozzino, 2007). While the PFC plays an important role in behavioral control in many situations, there are several other neural systems that also contribute unique features to decision-making and strongly influence choice (Balleine and O’Doherty, 2009; Dalley et al., 2011; Gruber and McDonald, 2012). Two pervasive choice strategies across species and tasks are lose-shift and win-stay responding, which have long been studied as measures of behavioral flexibility (Evenden and Robbins, 1983). These responses reflect the propensity of participants to repeat choices that resulted in a reward in the previous trial (win-stay), and to shift responding away from options that formerly led to a poor outcome (lose-shift). Humans show this type of behavior in various tasks and reward contingencies (Hayden and Platt, 2009; Worthy et al., 2013).

We have recently discovered (Skelin et al., 2014; Gruber et al., 2017) that lose-shift responding in rats depends on the lateral striatum (LS), a sensorimotor region of the striatum homologous to the putamen in primates (Johnston et al., 1990; Voorn et al., 2004; Balleine and O’Doherty, 2009). The PFC normally inhibits the behavioral control exerted by sensorimotor systems, which includes the expression of habits (Jahanshahi et al., 1998, 2000; Knoch et al., 2005). We therefore hypothesized that impairment or preoccupation of PFC will lead to increased control by sensorimotor systems, and therefore increased lose-shift responding in humans. The influence of executive systems in choice is reduced when participants are given cognitively demanding tasks to perform in tandem with decisions. A typical method is to have the participants perform a serial subtraction task in which they recite a numerically descending series (Brown et al., 1999; Ingram et al., 2000). Thus, we predicted that participants engaged in such cognitively demanding activity while performing the choice task will exhibit increased prevalence of lose-shift responding. We tested this prediction here by having participants engage in a competitive binary choice task (CBCT) with or without concurrent cognitive load.

We made a second prediction based on the fact that the PFC is not fully developed in humans until adulthood (Sowell et al., 1999; Fuster, 2002; Gogtay et al., 2004). It, therefore, likely has weaker control over sensorimotor and other brain systems in children (Zelazo et al., 1996, 2004; Munakata et al., 2012). We thus predicted that children without additional cognitive load would show lose-shift responding on the CBCT similar to the cognitively loaded adults. Indeed, we found here that children (5–9 years old) and cognitively loaded adults showed prevalent lose-shift responding, whereas adults with no additional cognitive load and pre-teens did not. Further, we provide strong evidence that the mechanisms underlying lose-shift responding are temporally brief and are dissociated from those mediating win-stay. Thus, lose-shift responding appears to be a default and reflexive response strategy in humans that is normally suppressed by executive functions.

## Materials and Methods

### Competitive Binary Choice Task: Box Format

All procedures were approved by the University of Lethbridge Human Subject Research Committee. All subjects gave written informed consent in accordance with the Declaration of Helsinki and all procedures are in compliance with the APA ethical standards. For Experiment 1, we recruited male (*n* = 6) and female (*n* = 12) participants (age 18–26) from the undergraduate student population at the University of Lethbridge. Participants provided informed consent after the nature and possible consequences of the studies were explained, and received course credit for their participation. We analyzed data from participants screening negative on written self-evaluations for Attention-Deficit Hyperactivity Disorder using the World Health Organization Adult Self-Report Scale, substance abuse (WHO-ASSIST v3.0), problem gambling (CAMH Gambling Screen), head injury requiring medical care, and prior anxiety/depression diagnosis.

The task was implemented on a touch-screen enabled tablet computer (Microsoft Surface Pro 3). Participants first touched a centrally-located square to initiate a trial, and then touched one of two target boxes that would then appear 3 cm on either side of the square. Immediately following, the target boxes disappeared, and a numeric value would appear to indicate either a win (“Win $10” in green text) or loss (“Lose $10” in red text). This message remained for a randomly chosen delay period (1–4.5 s). Afterwards, the screen went blank and the square would appear to initiate the next trial. The computer implemented a competitive zero-sum game sometimes called “Matching Pennies”, which has been previously described (Seo et al., 2007; Vickery et al., 2011; Skelin et al., 2014). Briefly, the algorithm attempted to minimize the number of rewards delivered. The algorithm would choose the rewarded side randomly, unless it detected that the subject was likely to non-randomly select a target option. The detection was done by computing the likelihood that a participant was engaging in patterned responses over the past 1–4 trials. If the previous sequence of responses to the right (R) and left (L) option (and reinforcement) was R(win), R(lose), R(win), R(lose), then the algorithm would compute whether the L side was likely to be selected with a probability greater or less than chance by computing frequency of the sequences RL, RRL, RRRL and RRRRL in all past trials in the session. It analogously computed the frequency of sequences of choice and reward (e.g., R(lose)L, R(win)R(lose)L, …). If any of these sequences occurred more than at chance levels (probability > 0.5 by the binomial test, *p* < 0.05), the algorithm would then select the L side to be unrewarded. The same procedure was used to determine if the participant was more likely than chance to select the R option, in which case it would be unrewarded on the current trial. If neither the R or L choice were more likely than chance, then the rewarded side was selected randomly. The competitive algorithm therefore punished predictable response patterns. The optimal strategy for the participants was to be as stochastic as possible in their choices. Participants performed 180 trials per session. Concurrently with the competitive choice tasks, some participants (*n* = 8) were instructed to also engage in a serial subtraction task as a cognitive load. The task consisted of reciting the numeric series starting with 999 and decrementing by 3 on each iteration. If participants reached the number 0, they were instructed to start over beginning with 998. If the recitation rate became lower than one number every 3 s, the experimenter asked them to try to count faster. One subject was unable to perform the counting task, and another subject generated lose-shift behavior that was 6 standard deviations from the population mean and was determined to be an outlier by the Extreme Studentized Deviate method; both participants were excluded from the data set. A total of 16 participants were thus included in the analysis. The inter-trial interval (ITI) was computed as the time from the onset of the feedback to the press of the central square to start the subsequent trial. We then ran a second experiment in which the feedback was presented for 1 s every trial, and a blank screen was presented for a delay of 1, 6.5 or 12 s (randomly chosen for each trial). All participants in this experiment completed the task concurrently with the cognitive load. We excluded participants that either could not perform the serial subtraction task (more than 20 errors in the session; *n* = 2), or employed a choice pattern independent of reinforcement (e.g., alternation; *n* = 2). A total of *n* = 12 participants were included in the analysis for Experiment 2.

### Competitive Binary Choice Task: Maze Format

For Experiment 3, we recruited male (*n* = 14) and female (*n* = 42) participants (age 18–27; mean age = 20.8, SD = 2.64) from the undergraduate student population at the University of Lethbridge. Subjects received course credit for participation. Consent and subject screening was performed as described for the Box format of the task. We implemented the same competitive algorithm described above on a touch-screen enabled tablet PC. In this format of the task, the participants had to choose between left and right targets by guiding a video character (a raccoon) to one of two trees in order to find a hidden target (a strawberry). The participants would use their finger to drag the raccoon icon on a touchscreen to either the right or left side of a partition. The tree disappeared upon character contact, and revealed either a strawberry (a win) or the background color (a loss). A progress bar at the top of the screen indicated the relative number of trials completed. Exclusion criteria were the same as previously described, resulting in elimination of participants that scored positive on the ADHD Self-Report Scale (*n* = 4), those who had a “moderate” or greater risk score on the WHO-ASSIST substance abuse questionnaire (*n* = 12), those who employed a strongly patterned response, such as alternation or perseveration on one side (*n* = 9), those diagnosed with anxiety/depression (*n* = 4), those who were significantly slower and were determined to be an outlier by the Extreme Studentized Deviate method (*n* = 1), or who could not perform the serial subtraction task (*n* = 1). Consistent with our previous design, some participants (*n* = 12) were instructed to do the counting task while performing the experiment. A total of 25 participants were included in the analysis for this group. All adults performed 180 trials on the task so as to be consistent with the box version of the task.

For Experiment 4, we recruited 17 children (nine female, eight male), with ages between 5 years and 9 years old (mean age = 7.4, SD = 1.04), to perform 40 trials of the same Maze format of the task described above. The children were enrolled through a local elementary school and parental consent was obtained. Parents were informed of the nature and possible consequences of the studies, and were present during testing. Participants were excluded if the parent reported a prior diagnosis of ADHD (*n* = 1), if they were significantly slower (*n* = 1), or if they used a strongly patterned response (*n* = 4). Therefore, 11 participants were included in the analysis. At the end of the session, the children received a small toy regardless of their performance.

For Experiment 5, 14 children (six female, eight male) with ages between 11 years and 13 years old (mean age = 12.1, SD = 0.94) were tested on the same task in the Maze format for 140 trials. Parents provided written consent and were given the option to attend the testing. Participants were excluded if they used a strongly patterned response (*n* = 1). All participants received a voucher for entry to a local movie theater after testing.

### Analysis

Data were analyzed and plotted with custom written code and built-in function of Matlab 2013a or GraphPad Prism version 7. The probability of lose-shift was calculated as the probability that the subject would chose the alternate response option in trials following reward omission. Likewise, the probability of win-stay was calculated as the probability that the subject would repeat the selection on trials immediately following rewarded trials. In defining consecutive trials, we include only trials that were less than 20 s apart. Mean values for each group were computed based on session-averaged data for each subject. When comparing among different cohorts of adults, all 180 trials were used for the session means. Because the young children only completed 40 trials, we generated session means for the adult contrast groups based on the first 40 trials of the session. The behavioral responses of adults computed over the first 40 trials was not different than that computed for the full session (*t*-tests, *p*’s = 0.09–0.89). Furthermore, analysis of behavior binned into quartiles of trial number within the session revealed no difference in either lose-shift (RM analysis of variances (ANOVAs), *p*’s = 0.14–0.99) or win-stay (RM ANOVAs, *p*’s = 0.29–0.99), regardless of the cognitive load condition. Data were normally distributed as determined by the D’Agostino-Pearson test in GraphPad Prism (alpha = 0.05) unless otherwise stated.

## Results

**Figure 1:**
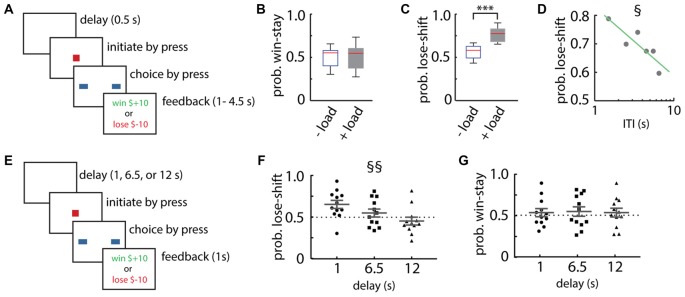
Lose-shift responding by healthy adult participants. **(A)** Schematic illustration of the Box format of the competitive binary choice task (CBCT). **(B,C)** Adult participants show little lose-shift or win-stay responding above chance level when performing the task with no other cognitive load (−load), but a prominent lose-shift strategy emerges in participants that are performing concurrently with a cognitively demanding serial subtraction task (+load). Box plots show the median (red line), upper and lower quartiles, and the extreme data points (whiskers). **(D)** The probability of lose-shift responses for the +load group, which decreased with increasing inter-trial interval (ITI). **(E)** Schematic illustration of the modified choice task with a blank-screen delay of 1, 6.5 or 12 s. **(F,G)** Participants showed reduced lose-shift with longer delays, whereas win-stay did not vary with delay; plots show individual values, mean and error bars represent SEM. Statistical significance (*p* < 0.001) is indicated by “***”. Main effect of ITI (*p* < 0.05) is indicated by § and (*p* < 0.00005) is indicated by §§.

We used a touch-screen tablet computer to assess choice strategies in adults and children performing the CBCT. The task had two presentation formats, a Box version in which the participants chose one of two rectangular boxes in order to collect a fictitious monetary reward, or a Maze version in which participants guided a cartoon character to one of two trees to find a concealed target. We first tested how adult participants adapted responses on trials immediately following wins or losses in the Box format (Figure 1A). This task is modeled after the classic “Matching Pennies” game in which two players compete by each making a binary choice, such as secretly turning a coin to be heads or tails. The rules for winning are established before play such that one player wins if both players’ choices match, and the other wins if the choices do not match. Previous studies have shown that humans approach the optimal solution against rational opponents, which is to select randomly on each trial to win on 50% of the trials (Vickery et al., 2011). This is because any predictability in the choice of the player, such as alternation, can be exploited by the opponent so that the player wins less than 50% of the time. Participants should therefore avoid using lose-shift or win-stay strategies in this task. Deviation from random responding reveals innate choice strategies. The opponent in the present work is a computer algorithm.

### Cognitive Load Increases Lose-Shift Responding in Adult Human Participants

Our first objective was to test our hypothesis that taxing the executive system with cognitive load would increase lose-shift responding. We therefore instructed one group to perform a cognitively demanding task (serial subtraction of 3 starting from 999) during the task (*n* = 8), while a control group did not have this additional cognitive load (*n* = 8). The control group did not exhibit lose-shift or win-stay strategies beyond that expected by chance (Figures 1B,C), consistent with the optimal strategy on the task. The cognitive load did not increase win-stay responding (*t*-test that mean is the same with or without load; *t*_(15)_ = 0.06, *p* = 0.48; Figure 1B), but did significantly increase lose-shift responding as compared to the control group (two-tailed *t*-test: *t*_(15)_ = 4.59, *p* = 2E-4, *d* = 1.77; Figure 1C). This suggests that lose-shift responding is normally suppressed by the executive systems preoccupied by the counting task, whereas win-stay is not.

We next sought to determine if the memory trace supporting lose-shift responses decays with time, as has been reported in rats (Gruber and Thapa, 2016). We found that the lose-shift probability among the group with the cognitive load does indeed decay with increasing (ITI; F vs. constant model = 13.4, df = 4, *p* = 0.02; *r*^2^ = 0.71; Figure 1D). The ITI is defined here as the time between the onset of reward feedback and the choice on the subsequent trial. In this experiment, the delay was randomly set between 1 s and 4.5 s by the software, but participants showed self-paced ITI spanning approximately 1–9 s. We thus ran a second experiment with new participants (*n* = 12) under cognitive load in which the delays (blank screen without feedback) were randomly presented within sessions at fixed intervals of either 1, 6.5, or 12 s (Figure 1E). This delay is exclusive of the reinforcement feedback (1 s) and the initialization time on the subsequent trial. These participants also exhibited strongly decreased lose-shift responding as the ITI increased (main within subjects effect of ITI RM-ANOVA: *F*_(2,22)_ = 17.1, *p* = 3E-5; Figure 1F), whereas the probability of win-stay was not affected by delay (main within subjects effect of ITI RM-ANOVA: *F*_(2,22)_ = 0.1, *p* = 0.93; Figure 1G). These data provide strong evidence that the memory trace supporting lose-shift responding decays over several seconds in humans.

### Children Perform Similarly to Adults Under Cognitive Load

**Figure 2:**
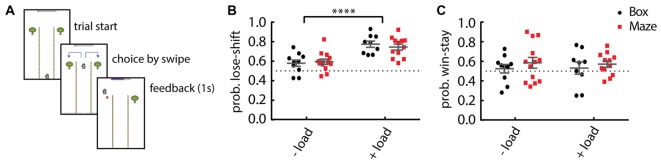
Comparison of performance among task formats. **(A)** Schematic illustration of the Maze format of the task. **(B)** Adult participants show little lose-shift responding when performing either task format (Box or Maze) under no other cognitive load (−load), but a prominent lose-shift strategy emerges when they concurrently engage in serial subtraction (+load) in either task format. **(C)** Win-stay responding is invariant to task format or cognitive load. Plots show individual values, mean and error bars represent SEM. Statistical significance (*p* < 0.0001) is indicated by “****”.

We hypothesized that the underdeveloped executive functions in children would result in increased lose-shift responding, even in the absence of additional cognitive load. Because pilot testing revealed that children were not engaged in the Box version of the task, we developed a more game-like version modeled after the classic T-Maze (Figure 2A). We first tested whether the change in task format significantly affected the performance of adults. We found no difference in performance on the Maze format as compared to the Box format for either adult control participants or adults under cognitive load. Regardless of the presentation design (multiple comparisons Two-way ANOVA; main effect of design: *F*_(1,40)_ = 0.0553, *p* = 0.81), the probability of lose-shift increased significantly when adult participants have an increased cognitive load (main effect of load: *F*_(1,40)_ = 32.1, *p* = 1.39E-6; Figure 2B). However, win-stay is not significantly dependent on the cognitive load (*F*_(1,40)_ = 0.005, *p* = 0.94; Figure 2C) or the task format (*F*_(1,40)_ = 1.038, *p* = 0.31).

**Figure 3:**
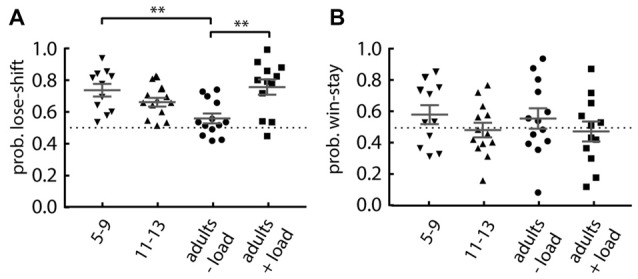
Performance of children compared to adults. **(A)** Lose-shift responding is significantly increased in 5–9 years old children and adults under cognitive load, compared to the (−load) adults. **(B)** The probability of win-stay in children is similar to that in adults, regardless of their cognitive load. Plots show individual values, mean and error bars represent SEM. Statistical significance (*p* < 0.005) is indicated by “**”.

We next investigated the performance of younger (aged 5–9) and older (aged 11–13) children on the Maze format of the task under no additional cognitive load. The younger children were asked to perform 40 trials, so we compared their performance to the first 40 trials of the comparison groups: older children; adults with no cognitive load; and adults with cognitive load. We found a main effect of group on lose-shift responding (one-way ANOVA: *F*_(3,45)_ = 6.02, *p* = 0.0015; Figure 3A). *Post hoc* tests revealed that when compared to the adults performing without the cognitive load, the younger children (Dunnett’s multiple comparison *post hoc* test: *p* = 0.004; *d* = 1.45) and the adults engaged in serial subtraction (*p* = 0.001; *d* = 1.39) show an increased lose-shift response. This effect disappears in the older children. The 11–13 years old show no difference (*p* = 0.119). There was no difference for win-stay responding for any group (one-way ANOVA; main effect of group: *F*_(3,45)_ = 0.793, *p* = 0.504; Figure 3B).

**Figure 4:**
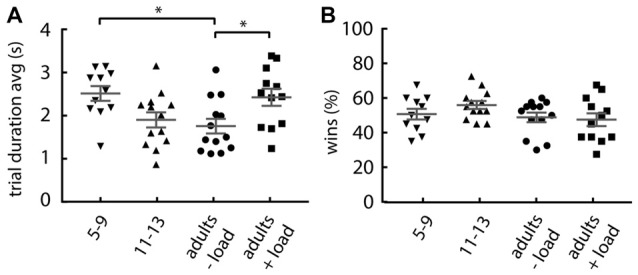
Effect of group on response rate and number of wins. **(A)** Mean trial duration, showing that adults under load and 5–9 year olds tend to be slower. **(B)** Percentage of rewarded trials, showing that each group collected equivalent proportions of wins. Plots show individual values, mean and error bars represent SEM. Statistical significance (*p* < 0.05) is indicated by “*”.

Given that the probability of lose-shift reduces with slower performance (Figure 1D), the increase in lose-shift under cognitive load could be explained if participants increased their performance speed when counting. However, the groups with increased lose-shift responding have an increased mean trial duration over the first 40 trials (one-way ANOVA; main effect of group: *F*_(3,45)_ = 4.366, *p* = 0.008; Figure 4A). The 5–9 years old and cognitively loaded adults tend to be slower than the non-counting adults (Dunnett’s multiple comparison *post hoc* test; children: *p* = 0.012, *d* = 1.28; +load adults: *p* = 0.028, *d* = 1.02). The results of Experiment 1 indicate that the slowing effect of the load or young age should *decrease* lose-shift, and thus does not account for the observed increase. In addition, the number of wins was not affected by load or age (one-way ANOVA: *F*_(3,45)_ = 1.61, *p* = 0.199; Figure 4B), which indicates that the increase in lose-shift in the +load and 5–9 years old groups is not due to an overall performance deficit, frustration, or other factor related to a different frequency of wins and losses.

## Discussion

We investigated decision-making processes in adults and children, using a deceptively simple task in which random responding is the best selection policy. Deviation from random responding reveals innate choice strategies. The data show that reward omission has a pronounced short-lasting effect on subsequent choice, which can be described by the classic notion of lose-shift responding. We found here that lose-shift is prominent in children and in adults under cognitive load, but not in older children or adults unburdened by other cognitively effortful tasks.

Humans show win-stay/lose-shift (WSLS) responses in several behavioral contexts. Participants rely heavily on a WSLS strategy in other binary and trinary analogs of the present task (Vickery et al., 2011). Choice behavior on the Iowa Gambling Task (IGT) is also well explained by a WSLS strategy (Worthy et al., 2013). This tactic yields superior results in some games such as the Prisoner’s Dilemma (Nowak and Sigmund, 1993; Posch, 1999), but not the present task. The competitive nature of our task ensured that high levels of predictable strategies diminished the number of rewards. Nonetheless, while control participants employed little to no lose-shift, participants performing under increased cognitive load continued to employ it throughout the session. Together, these studies indicate that lose-shift responding should be considered when interpreting data from choice tasks.

The probability of lose-shift responding decayed with increasing delays between the feedback signal and the next choice. This decay is very similar to that of the lose-shift observed in rodents performing against the same computer algorithm (Gruber and Thapa, 2016). The time dependence is consistent with findings in other species. WSLS responding decreases with longer ITIs in rhesus monkeys (Deets, 1970) and in pigeons (Rayburn-Reeves et al., 2013). We did not find such a temporal relationship with win-stay in the present data, providing evidence supporting the hypothesis that win-stay and lose-shift are mediated by different neural mechanisms (Skelin et al., 2014; Gruber and Thapa, 2016; Gruber et al., 2017). We can only speculate on the information encoded by the decaying memory trace, but we suspect it is an inhibition of the reward position rather than an explicit representation of the reward omission. We base this on a separate set of experiments (unpublished data) in which the targets (box version) appeared in a translated position on some trials. The probability of lose-shift was lower when the target pairs shifted in space as compared to when they were presented in the same location as in the previous trial. This should not occur if the memory was based primarily on reinforcement and not position.

Although we cannot rule-out the possibility that the memory trace supporting lose-shift involves working memory, we argue that this is unlikely for several reasons. Lose-shift prevalence *increases* under cognitive load. The serial subtraction task we used appears to involve the dorsolateral PFC (Burbaud et al., 1995; Vansteensel et al., 2010). This structure is also proposed to subsume working memory (Berman et al., 1995; Curtis and D’Esposito, 2003). It thus stands to reason that the cognitive load should reduce lose-shift responding if the response depends on working memory. However, we acknowledge that the complex nature of working memory and its temporal variability makes it difficult to rule out. It is possible that the limitations of the executive function in the cognitively loaded adults and young children has constrained the working memory to a shorter time span such that choice is based more prominently on the immediately previous trial, rather than some weighted average of past outcomes.

We instead propose that lose-shift is mediated by sensorimotor systems, including the putamen in primates or LS in rodents. This is consistent with lesion data in rats (Skelin et al., 2014; Gruber et al., 2017), and impairments in a similar task (Rock-Paper-Scissors) in human patients with damage to the putamen (Danckert et al., 2012). Furthermore, it provides a parsimonious explanation for the increase in lose-shift in adults under load: the normal suppression of sensorimotor control by PFC (Jahanshahi et al., 1998; Knoch et al., 2005) is disrupted by the subtraction task, thereby unmasking lose-shift behavior mediated by sensorimotor system. This also provides an explanation for the ubiquity of lose-shift responding across an enormous variety of animals, including pigeons (Rayburn-Reeves et al., 2013), mice (Amodeo et al., 2012), rats (Evenden and Robbins, 1983), and monkeys (Lee et al., 2004). The sensorimotor systems, including the striatum, are largely phylogenetically conserved in mammals (Johnston et al., 1990). If lose-shift is mediated by this conserved structure, we expect it to present in many species. We propose that the reason rats lose-shift more on the analogous task (68 ± 1%; Gruber and Thapa, 2016) is that they have a more primitive PFC that does not hold the same level of cognitive control over sensorimotor systems, leaving them to rely more on this reflex responding.

We cannot rule out possible PFC contributions to performance other than sensorimotor suppression. The PFC mediates many computations used in decisions. For instance, humans with lesions in the ventromedial PFC (vmPFC) show impaired decisions involving future consequences (Bechara et al., 1994), and the orbitofrontal region of PFC appears to be important for decisions based on reward magnitude (Rogers et al., 1999). The task used here, however, does not require such computations. The vmPFC does activate during guessing (Elliott et al., 1999), which may be relevant in our task. On the other hand, another study showed no activation of the PFC during WSLS responses in a two-choice prediction task using fMRI (Paulus et al., 2001). This latter study is consistent with our proposal that WSLS is mediated predominantly by the striatum.

The dorsolateral PFC (dlPFC) is likely involved in performance on the present task. The cognitive control mediated by this region is exerted through both proactive and reactive strategies (Paxton et al., 2008; Braver et al., 2009). For instance, it exerts behavioral proactive inhibition via the putamen (Smittenaar et al., 2013). Furthermore, the dlPFC-dorsal striatum circuit is activated when participants are suppressing the tendency to claim an immediate reward in lieu of a larger delayed one (Tanaka et al., 2006). This circuit is engaged when participants need to employ a strategy based on past choices and reinforcements to maximize their rewards. Conversely, when previous decisions have no influence over the outcome, the OFC-ventral striatum loop is activated (Tanaka et al., 2006). These results support the hypothesis that the PFC normally inhibits the reflex of lose-shift, but when it is engaged in another highly demanding task, it releases the brake on the sensorimotor system and allows it to exert control over behavior.

The relatively late development of cognitive control in young children has been linked to the late neurobiological development of the PFC, which likely accounts for their relatively poor performance in various tasks as compared to adults (Diamond, 1988; Zelazo et al., 1996; Hooper, 2004). Children have deficits in inhibiting inappropriate responses, which correlate with a lack of activation of the PFC (Bunge et al., 2002). Inadequate response inhibition, along with working memory deficits, have been reported in adolescents (ages 9–17) performing the IGT (Hooper, 2004). In another study, however, healthy 12–14 year olds are reported to perform similarly to healthy adults in the IGT (Ernst et al., 2003). Such discrepancies likely involve the ongoing, and highly variable, development of PFC prior to adulthood (Adleman et al., 2002). In our task, the 11–13 year old group performs at an intermediary level between the control adults and the 5–9 year olds. We acknowledge that our relatively small sample size does not allow us to make any definitive statements, but we speculate that the data reflects that the level of development of executive functions in the older children surpasses the one of the 5–9 year old group.

In addition to cognitive control, the development of the human brain also affects systems involved in learning from reinforcements. A recent study examined the functional connectivity between the medial PFC and the striatum in participants between the ages of 8 and 22 performing a reinforcement learning task (van den Bos et al., 2012). They found that as age progresses, participants are less influenced by negative feedback, suggesting that adults utilize lose-shift responding less than children. These differences in adaptive learning are correlated with the strength of the functional connectivity between the ventral part of the putamen and mPFC. This corroborates our findings.

In conclusion, lose-shift responding appears to be a reflexive response strategy in humans that is normally suppressed by executive functions of the PFC. It is easily measured with little or no subject awareness, and may be a useful metric for determining the governance of PFC in decisions. Lose-shift responding has a prominent effect on trial-by-trial choice adaptation, particularly when ITIs are short and executive systems are otherwise occupied or impaired. It is therefore an important component to include in theories and computational models of choice, and a potential confound in behavioral experiments in humans.

## Author Contributions

VEI was involved in all parts of the project, with support from PJB for data collection and software design, KG for data collection, and with substantial input from AJG for data analysis and writing the manuscript. PJB is now at McMaster University, ON, Canada.

## Conflict of Interest Statement

The authors declare that the research was conducted in the absence of any commercial or financial relationships that could be construed as a potential conflict of interest.
